# The Effectiveness of High-frequency Repetitive Transcranial Magnetic Stimulation in Persistent Somatoform Pain Disorder: A Case Series

**DOI:** 10.7759/cureus.2729

**Published:** 2018-06-01

**Authors:** Shubh Mohan Singh, Vijay Prakash, Swati Choudhary, Ajit Avasthi

**Affiliations:** 1 Psychiatry, Postgraduate Institute of Medical Education and Research, Chandigarh, IND

**Keywords:** pain, somatoform, disorder, neuromodulation, repetitive transcranial magnetic stimulation, chronic

## Abstract

Somatoform pain disorders (SPD) are common, disabling and do not respond well to existing treatment modalities. We investigated the usefulness of 18 sessions of high frequency repetitive transcranial magnetic stimulation delivered at the left dorsolateral prefrontal cortex (DLPFC) in five right-handed patients with SPD. All patients reported significant improvement in pain relief and activities of daily living. High-frequency repetitive transcranial magnetic stimulation (rTMS) delivered at the left DLPFC may be useful in SPD.

## Introduction

Somatoform disorders including somatoform pain disorders (SPD) are among the most common psychiatric disorders and are frequently comorbid with depressive disorders [1]. They are generally recalcitrant and lead to significant dysfunction [1]. Various treatment modalities have been used in SPD but there is little evidence for effective treatments in the same [2]. Repetitive transcranial magnetic stimulation (rTMS) is useful in a variety of chronic painful conditions [3]. We hypothesised that high-frequency (HF) rTMS delivered over the prefrontal cortex (PFC) may be useful in SPD. This is because SPDs are frequently comorbid with depressive disorders and HF rTMS is effective in the latter [4]. Secondly, PFC has been shown to be involved in the pathogenesis of SPD [5]. Finally, PFC HF rTMS has been shown to be effective in reducing pain associated with resistant depression [3]. This study represents a preliminary investigation into the potential effectiveness of HF rTMS in SPD.

## Case presentation

The study protocol was passed by the institute ethics committee and the study was carried out in the rTMS service of the department of psychiatry of a tertiary hospital in North India. A prospective, open-label design was adopted. Purposive sampling was used to recruit five patients of either gender and aged at least 18 years referred to the service with a diagnosis of persistent somatoform pain disorder (F45.4) as per the ICD-10. All the patients were referred for rTMS treatment because of inadequate response to conventional treatment (mostly pharmacological). All patients provided written informed consent. History and clinical assessment was reviewed and diagnosis was confirmed. All patients were treated with rTMS as per a commonly used protocol [6]. The F3 location on the scalp was determined as per the 10-20 system. This location generally corresponds to the left dorsolateral prefrontal cortex (DLPFC). Motor threshold was detected using the visual method as per the intensity required to evoke contraction of the Abductor pollicis brevis muscle of the right hand at least 50% of the times the stimulus was delivered. The pulses were delivered at 100% of the motor threshold. The rTMS was delivered in form of pulses delivered at a frequency of 10 Hz for 4 seconds followed by a wait period of 26 seconds (high frequency). We delivered 1200 pulses/session (30 trains/session). One session/day was delivered each working day (Monday-Saturday). We delivered 18 sessions/patient. All treatments were delivered using a figure-of-8 coil on a Magstim Rapid2 machine. All patients continued to be managed by referring clinicians who could alter pre-existing prescriptions as per requirement. Assessments were carried out at baseline, weekly thereafter until the end of 18 sessions and finally at two weeks after last rTMS session. Pain assessment was done using a 10-cm visual analog scale (VAS). Table 1 presents the patient socio-demographic and clinical profiles and details of response to rTMS.

**Table 1:** Patient profile and response to rTMS. VAS: Visual analog scale; rTMS: Repetitive transcranial magnetic stimulation.

S. No.	Patient profile	Duration of symptoms in years	Current prescription	Comorbid psychiatric diagnosis	VAS Score					% decrease
					Baseline	Week 1	Week 2	Week 3	Week 5	
1	40 years old married female, illiterate, homemaker, pain over head, both upper limbs and trunk	10 years	Duloxetine 60 mg/day	None	8	8	7	6	6	25
2	50 years old married female, homemaker, illiterate, pain over head, neck and trunk	2 years	Duloxetine 60 mg/day	None	8	8	7	3	3	62.5
3	32 years old married male, 10 years of education, farmer, pain all over body	15 years	Venlafaxine 150 mg	Dysthymia (F34.1)	8	8	6	3	3	62.5
4	38 years old married female, illiterate homemaker, pain over head, abdomen	5 years	Venlafaxine 150 mg Duloxetine 60 mg	None	8	8	6	3	3	62.5
5	43 years old married female, homemaker, illiterate, pain over right side of body	18 years	Duloxetine 60 mg	Dissociative motor disorder (F44.4)	9	9	3	3	2	77.77

All patients were right-handed. None of the patients had any diagnosable physical illness. All patients were able to complete 18 sessions. No serious side-effects were noted or reported.

Our results indicate that HF rTMS delivered over the left DLPFC was effective in our cohort with SPD. The improvement started by the second week (after six sessions) and continued over the rest of the duration of rTMS. Improvement was sustained at two weeks after last session of rTMS. All patients also reported subjective improvements in activities of daily living and that the pain was reduced and bothered them a lot less than before. All patients were satisfied with the treatment. These results are in keeping with other reports that have shown the effectiveness of rTMS in chronic pain [3]. Our study is perhaps the first to report this in SPD.

## Discussion

SPDs are generally poorly researched and have low quality evidence with regards to the effectiveness of treatment options [1]. Both pharmacological and non-pharmacological options have been tried without much success and given the heterogeneity of clinical presentations, specific treatment guidelines are unavailable [7,8]. Whereas there is some evidence with respect to the effectiveness of rTMS in pain associated with depressive disorders and disorders such as fibromyalgia or neuropathic pain, to the best of our knowledge no such evidence exists for SPD [9]. Various mechanisms for the putative effectiveness of rTMS in such conditions have been postulated but the exact mechanisms remain unknown. In such a scenario, treatment options such as rTMS in SPD need to be investigated more thoroughly.

Our study found that HF PFC rTMS was effective in alleviating pain in SPD. The speed of improvement was similar to that has been reported in other trials for chronic pain due to fibromyalgia [10]. Thus similar mechanisms such as endogenous opioid release and functional improvements in brain areas associated with pain may be involved [9]. We also found that our patients reported a greater quantum of improvement than has been reported elsewhere. For instance, in a study on medically unexplained pain in patients with resistant depression, the improvement reported was 37.5% [11]. In our study, except for one patient all patients reported an improvement of at least 62.5%. These results must be seen in context of socio-educational background of the patients. It is well known that rTMS is associated with significant placebo response including in pain disorders [12]. It is likely that in our study as well, placebo response may have played an important part in the reported improvement.

## Conclusions

Our study indicates that rTMS needs to be investigated more thoroughly as a treatment option in SPD. This is important because existing treatment options in SPD are of limited utility.

## References

[1] Bass C, Peveler R, House A (2001). Somatoform disorders: severe psychiatric illnesses neglected by psychiatrists. Br J Psychiatry.

[2] Kroenke K (2007). Efficacy of treatment for somatoform disorders: a review of randomized controlled trials. Psychosom Med.

[3] Goudra B, Shah D, Balu G, Gouda G, Balu A, Borle A, Singh PM (2017). Repetitive transcranial magnetic stimulation in chronic pain: a meta-analysis. Anesth Essays Res.

[4] Lefaucheur J-P, André-Obadia N, Antal A (2014). Evidence-based guidelines on the therapeutic use of repetitive transcranial magnetic stimulation (rTMS). Clin Neurophysiol.

[5] Valet M, Gündel H, Sprenger T (2009). Patients with pain disorder show gray-matter loss in pain-processing structures: a voxel-based morphometric study. Psychosom Med.

[6] George MS, Lisanby SH, Avery D (2010). Daily left prefrontal transcranial magnetic stimulation therapy for major depressive disorder: a sham-controlled randomized trial. Arch Gen Psychiatry.

[7] van Dessel N, den Boeft M, van der Wouden JC (2014). Non-pharmacological interventions for somatoform disorders and medically unexplained physical symptoms (MUPS) in adults. Cochrane Database Syst Rev.

[8] Kleinstäuber M, Witthöft M, Steffanowski A, van Marwijk H, Hiller W, Lambert MJ (2014). Pharmacological interventions for somatoform disorders in adults. Cochrane Database Syst Rev.

[9] Li CT, Su TP, Hsieh JC, Ho ST (2013). Efficacy and practical issues of repetitive transcranial magnetic stimulation on chronic medically unexplained symptoms of pain. Acta Anaesthesiol Taiwan.

[10] Galhardoni R, Correia GS, Araujo H (2015). Repetitive transcranial magnetic stimulation in chronic pain: a review of the literature. Arch Phys Med Rehabil.

[11] Li C-T, Lin W-C, Chen M-H, Su T-P, Bai Y-M, Hsieh J-C (2016). Analgesic effect of repetitive transcranial magnetic stimulation on medically unexplained pain in patients with medication-resistant depression. Taiwan J Psychiatry.

[12] André-Obadia N, Magnin M, Garcia-Larrea L (2011). On the importance of placebo timing in rTMS studies for pain relief. Pain.

